# RETRACTED: Castellano-Tejedor, C.; Cencerrado, A. Gamification for Mental Health and Health Psychology: Insights at the First Quarter Mark of the 21st Century. *Int. J. Environ. Res. Public Health* 2024, *21*, 990

**DOI:** 10.3390/ijerph23050683

**Published:** 2026-05-21

**Authors:** Carmina Castellano-Tejedor, Andrés Cencerrado

**Affiliations:** 1 Psynaptic, Psicología y Servicios Científicos y Tecnológicos S.L.P, Sant Quirze del Vallès, 08192 Barcelona, Spain; acence@gmail.com; 2 GIES Research Group, Basic Psychology Department, Universitat Autònoma de Barcelona (UAB), 08192 Bellaterra, Spain; 3 Research Group on Aging, Frailty and Care Transitions in Barcelona (REFiT BCN), Parc Sanitari Pere Virgili & Vall d’Hebron Research Institute (VHIR), 08023 Barcelona, Spain; 4 Department of Psychology and Educational Sciences, Universitat Oberta de Catalunya (UOC), 08018 Barcelona, Spain

The journal retracts the article titled “Gamification for Mental Health and Health Psychology: Insights at the First Quarter Mark of the 21st Century” [1], cited above.

Following publication, concerns were brought to the attention of the Editorial Office regarding the accuracy of several references cited in this paper [1].

In accordance with the journal’s standard procedures, the Editorial Office and Editorial Board conducted an investigation that confirmed the presence of a significant number of non-existent references, which were determined to undermine the overall reliability of this study. In addition, the investigation identified indications of generative AI use in the preparation of the manuscript, without appropriate disclosure, as required by MDPI’s GenAI policy. 

Following the investigation, the Editorial Board determined that the extent of the issues identified did not support issuing a correction and decided to retract this publication [1], as per MDPI’s retraction policy.

This retraction was approved by the Editor-in-Chief of the *International Journal of Environmental Research and Public Health*.

The authors have been informed of this retraction and have indicated their agreement with this decision. The authors cooperated fully with the investigation and stated that they used automated tools to assist with the retrieval of bibliographic metadata during manuscript preparation.
